# Parents’ Experiences of Receiving a Severe Combined Immunodeficiency (SCID) or Non-SCID T-Cell Lymphopenia Outcome During the Newborn Screening Evaluation in England

**DOI:** 10.3390/ijns12020034

**Published:** 2026-05-12

**Authors:** Pru Holder, Chloe Musa, Jim B. Chilcott, Anju D. Keetharuth, Louise Moody, Ellinor K. Olander, Fiona Ulph, Jane Chudleigh

**Affiliations:** 1 Cicely Saunders Institute of Palliative Care, Policy and Rehabilitation, King’s College, London SE5 9PJ, UK; pru.holder@kcl.ac.uk; 2 Division of Psychology & Mental Health, Manchester Centre for Health Psychology, School of Health Sciences, Faculty of Biology, Medicine and Health, University of Manchester, Manchester Academic Health Science Centre, Oxford Road, Manchester M13 9PL, UK; chloe.musa2@nhs.net (C.M.); fiona.ulph@manchester.ac.uk (F.U.); 3 Sheffield Centre for Health and Related Research, University of Sheffield, Sheffield S1 4DA, UK; j.b.chilcott@sheffield.ac.uk (J.B.C.); d.keetharuth@sheffield.ac.uk (A.D.K.); 4 Centre for Arts and Creative Cultures, Coventry University, Coventry CV1 5FB, UK; l.moody@coventry.ac.uk; 5 Centre for Maternal and Baby Health Research, School of Health and Medical Sciences, City St George’s, University of London, London EC1V 0HB, UK; ellinor.olander.1@city.ac.uk

**Keywords:** severe combined immunodeficiencies, SCID, T-cell lymphopenia, non-SCID TCL, newborn bloodspot screening

## Abstract

Background: In 2021, the UK National Screening Committee commissioned an evaluation of newborn bloodspot screening for severe combined immunodeficiency (SCID) in England. This paper describes the experiences of parents who received an SCID or non-SCID T-cell lymphopenia (non-SCID TCL) result for their baby during the evaluation. Methods: A qualitative exploratory design was employed using semi-structured interviews with 12 parents (*n* = 5 who had received an SCID outcome and *n* = 7 who had received a non-SCID TCL following SCID NBS). Results: The impact on parents whose baby was diagnosed with SCID was complex, reflecting the experience of receiving a presymptomatic diagnosis. Parents of babies who had been diagnosed with a non-SCID TCL viewed their baby’s result in terms of risk; while their baby might still have a serious immunological condition, it was not considered to be as serious as SCID. All parents reported that they valued their participation in the SCID screening evaluation. Conclusions: Support for families following a positive screening result for SCID needs to be considered. This includes tailored psychosocial support, given their experiences will not be the same as those of parents of non-screened babies with SCID.

## 1. Introduction

As of June 2024, screening for T-cell deficiency had been introduced in 42 countries worldwide [1]. In 2021, the UK National Screening Committee (UKNSC) recommended an evaluation of the potential introduction of NBS for severe combined immunodeficiency (SCID) in England [2]. During the evaluation, two-thirds of babies born in England were offered screening for SCID between September 2021 and March 2024. The SCID evaluation therefore enabled the comparison of outcomes, costs of care, and impact on parents between those whose babies had been screened and the rest of the UK [2].

SCID refers to a group of rare inherited disorders characterized by a profound deficiency of T-lymphocytes [3]. The overall incidence is estimated to be approximately 1 in 50,000 live births, although regional variation has been reported [4,5]. Individuals affected by SCID are highly susceptible to severe, life-threatening infections; babies identified with SCID require isolation. SCID is primarily treated with haematopoietic stem cell transplantation (HSCT), with thymic transplantation or gene therapy used less commonly. Without treatment, babies with SCID will usually die before they reach their first birthday.

During the evaluation, the dried blood spot sample routinely collected by midwives from babies at five days of age as part of the NBS Programme in England was used to quantify T-cell receptor excision circles (TRECs) [6]. While low TRECs suggest an increased risk of SCID, measurement of TRECs is not specific and may also detect a range of non-SCID T-cell lymphopenias (non-SCID TCL), e.g., ataxia-telangiectasia, the CHARGE syndrome, 22q11.2 deletion (DiGeorge) syndrome [7,8,9,10,11] idiopathic TCL, and non-syndromic TCL. Treatment for non-SCID TCL therefore depends on the underlying cause and is generally deemed to be less urgent than treatment for SCID. Treatment for non-SCID TCL may range from monitoring +/− prophylactic antibiotics (e.g., idiopathic non-SCID TCL) to thymic or HSCT transplant. While some non-SCID TCLs may have a less favourable prognosis than SCID, UK data indicate that HSCT outcomes for SCID remain excellent and generally exceed those for other inborn errors of immunity. Therefore, although not the targeted population for SCID NBS, babies identified with a non-SCID TCL diagnosis, may benefit from early recognition and subsequent treatment.

Through the evaluation period, parents of babies with low TRECs were informed via telephone by a member of the immunology team and invited to attend immunology centres throughout England, usually the next day, for confirmatory testing using flow cytometry. Flow cytometry results were usually available the same day. These were explained to parent(s) and depending on the result, they were either discharged (false positive NBS result) or appropriate management or follow up investigation were arranged if a clinically significant outcome was identified (SCID or non-SCID TCL). Therefore, regardless of the diagnostic outcome, the SCID NBS journey was similar, but the treatment pathway differed depending on whether the baby was diagnosed with SCID or non-SCID TCL. This paper describes the experiences of parents who received a SCID or non-SCID TCL diagnosis for their baby during the SCID NBS evaluation in England.

## 2. Materials and Methods

A qualitative exploratory design was employed using semi-structured interviews with parents whose baby received a SCID or non-SCID TCL result following NBS for SCID. This study was part of a larger programme of work approved by North West-Greater Manchester East Research Ethics Committee (reference: 21/NW/0360).

### 2.1. Setting

The six NBS laboratories (which sent NBS results to 13 National Health Service Trusts in England) that were involved in the SCID evaluation were invited to take part in the study. The evaluation took place between 6 September 2021 to 1 March 2024.

### 2.2. Inclusion Criteria

Parents (defined in this context as individuals with parental responsibility, including legal guardians, as defined under the Children Act 1989 [12]) of babies who had been screened during the SCID NBS evaluation, aged over 18 years, who had received a SCID or non-SCID TCL result for their baby and were deemed to be able to understand the purpose and implications of the research study, were eligible for inclusion.

### 2.3. Recruitment and Sampling

A member of the immunology team who cared for the family when they attended for flow cytometry following the positive NBS result for SCID provided parents with a study “postcard” and/or a Participant Information Sheet (PIS). Parents could either contact the research team directly using the details provided in the PIS, consent to their contact details being shared with the research team or return the postcard. Returning the postcard with contact details indicated consent to be sent the relevant PIS with further details of the study. Postage-paid envelopes were supplied, and each postcard included a form allowing parents to request the information in a different language or format. Parents who returned a postcard or who agreed to share their contact details with the research team were then contacted and asked whether they were willing to take part in an interview. Parents were invited to take part in an interview no sooner than three months after receiving their baby’s NBS result, with follow-up interviews planned to take place annually on or around the baby’s birthday until five years of age.

### 2.4. Interviews

The interview schedules were developed by researchers with expertise in qualitative methods in the field of NBS, in collaboration with an oversight group comprising five stakeholders, including professionals and parent representatives. The oversight group met prior to the start of the project to review and provide feedback on the proposed data collection approaches and interview techniques.

Interviews took place online via Microsoft Teams (via Microsoft 365). During the interviews, researchers (JC, PH, CM) used a set of predefined topic areas, including the impact of screening on parents, parents’ views of their babies following the screening result, and their emotional and care journeys. To explore their emotional and care journey in more depth, the interviews incorporated a technique known as journey mapping, a design tool used to capture healthcare experience from the patient or service user’s perspective by mapping key ‘touch points’ with services [13,14,15]. This technique has been used previously in studies exploring parents’ experiences of NBS [16]. The order and precise wording of questions were adapted in response to participants’ answers to elicit richer and more detailed insights.

At the end of the interview, parents were asked demographic questions to enable the sample to be described based on items used in previous qualitative studies exploring families’ experiences of NBS [17,18]. These questions captured information such as language spoken at home, age, ethnicity, and family structure.

Written informed consent was obtained from all participants prior to the interviews. Parents were debriefed at the end of each interview to minimize potential distress. Participants were given the name and telephone number of a member of the research team whom they could contact at any time should they have had any concerns following their participation. Interviews were audio recorded and the transcription function in Microsoft Teams was used to produce verbatim transcripts that were checked for accuracy by the relevant interviewer.

### 2.5. Data Analysis

A deductive thematic analysis was conducted using a codebook, which was iteratively refined as the analysis progressed. Themes were generated using a latent approach, involving the interpretation of the underlying and implicit meanings in parents’ accounts [19]. Following familiarization with the data, two interview transcripts were coded independently by six members of the research team (CM, EKO, FU, JC, LM, PH) to support coding comparison and to inform and align the early stages of code development [20]. A codebook was then constructed based on these jointly coded transcripts. The same six researchers subsequently coded an additional transcript using the draft codebook, after which coding was compared to further refine and confirm code definitions. Remaining interviews were then coded by the three members of the research team (JC, PH, CM).

All extracts relating to each code and theme were collated to support theme development. Coding and theme generation were iterative processes, with new codes added and definitions refined as analysis progressed between the three coders ((JC, PH, CM). Once initial coding was complete, the final themes were reviewed and refined.

Demographic data were analyzed and summarized using simple descriptive statistics to describe the sample.

## 3. Results

### 3.1. Sample and Demographics

Of the 955,507 babies screened during the evaluation, 12 babies with SCID were identified and 56 babies with a non-SCID TCL diagnosis. Of these, we recruited five parents (two mothers and three fathers) of three (25%) babies with SCID. We also recruited seven (five mothers and two fathers) parents of six babies (11%) who received a result indicating their baby had a non-SCID TCL diagnosis. No deaths occurred among the infants with SCID or non-SCID TCL during the study. Parents were recruited from six of the National Health Service Trusts included in the evaluation.

All parents (*n* = 5) of babies with SCID were married or in a partnership with a co-parent, and all were employed. Parents were aged between 31 and 50 years. Four parents described their ethnicity as English/Welsh/Scottish/Northern Irish/British and reported English as their first language. One parent identified their ethnicity as African and their first language as non-English but stated that they did not require a translator for the interview. Four parents were educated to degree level or above, and one reported having ‘other qualifications’.

Six parents of babies with non-SCID T-cell lymphopenia were married or in a partnership with a co-parent, one was separated from a co-parent. Three were employed, two were looking after home/family, and two were on maternity leave. Parents were aged between 18 and 40 years. All (*n* = 7) described their ethnicity as English/Welsh/Scottish/Northern Irish/British and reported English as their first language. Three parents were educated to degree level or above, three had AS-, A-level equivalents (UK qualifications typically completed at ages 16–18, comparable to upper secondary education in other countries) and one had undertaken an apprenticeship.

### 3.2. Interview Data

Between June 2023 and December 2024, a total of 16 interviews were undertaken; six of these were follow-up interviews following the initial interview. Three were joint interviews with the baby’s mother and father, and the remainder were undertaken separately; eight with the baby’s mother and five with baby’s father. Initial interviews ranged in length from 40 to 106 min, with a median of 55 min. Follow-up interviews ranged in length from 21 to 57 min, with a median of 34 min. The intention was to undertake an initial interview no sooner than three months after receiving their baby’s NBS result, and follow-up interviews annually on the baby’s birthday until five years of age. However, when contacted, several parents stated they were reluctant to be interviewed until after their baby had received treatment for their condition. This was particularly true for parents of babies with SCID; consequently, the median age of babies with SCID at first interview was higher than babies with non-SCID T-cell lymphopenia (20 months and four months respectively). The length of interviews and ages of all babies are detailed in Table 1.

### 3.3. Themes: Initial Interview with Parents

The following nine themes were derived from the initial interviews with parents following their baby’s SCID or non-SCID TCL outcome.

#### 3.3.1. Confronting Risk in the Context of a Seemingly Healthy Infant

While many parents recalled that their baby had been screened, they had assumed at the time that their baby was healthy. One parent had not even considered the possibility of a positive NBS result.

“…you just don’t even think about what could happen as a consequence of those test results and you’re probably not even listening, because you don’t expect anything to be wrong…”

P18, Mum, Site 5, SCID, 13 months

Parents in both diagnostic groups (SCID and non-SCID TCL) reflected that their experience of finding out their baby was at increased risk of having SCID had challenged them to reframe their previously held beliefs about risk and vulnerability.

In addition, parents of babies who were subsequently diagnosed with SCID found it hard to reconcile how, with no known family history of genetic variants, their seemingly healthy baby could be at risk of a potentially life-threatening condition if not detected and treated promptly.

“I didn’t know I was a carrier of SCID, you know, we’d never had any family deaths, no baby deaths. Completely unaware, you know, you go to have a baby, and you don’t know what you carry.”

P45, Mum, Site 4, SCID, 28 months

For another parent, their own good health, coupled with the fact their baby had been asymptomatic, meant they had not contemplated the possibility of anything being detected via NBS.

“I didn’t take it seriously enough ’cause my default assumption was everything is going to be fine. Like, she looks healthy, [mum] and I are both healthy.”

P68, Dad, Site 6, Non-SCID TCL, 3 months

This prompted parents to reconsider aspects of their baby’s development, allowing them to attribute the abnormal result to a potential error in the NBS test rather than a problem with their baby.

This mismatch between appearance and potential severity sometimes led to initial denial, with parents attributing the result to testing error rather than potential illness. It also led to self-doubt and one parent of a baby diagnosed with non-SCID TCL questioning their competence, particularly their ability to detect when their baby might be unwell.

Due to this disconnect between the baby’s appearance and their NBS result, parents of babies in both diagnostic groups (SCID and non-SCID TCL) were shocked to receive an outcome suggesting that their baby might be at increased risk of having SCID. This manifested in a range of emotions upon receiving their baby’s positive NBS result, with parents describing the experience as traumatic and leading to a sense of loss of reality.

One parent whose baby was later diagnosed with non-SCID TCL, attributed some of their fear, before diagnostic testing and prior to a definitive outcome being known, to the potential treatment pathway for SCID. This was further exacerbated by the physical and emotional demands of the early postnatal period.

“…when I was told what [SCID] was, that it meant that she would have no immune system…she would need a bone marrow transplant…honestly my heart just was in my throat…it was petrifying hearing that…especially when you’re a brand new mum…and, sleep deprived.”

P74, Mum, Site 2, Non-SCID TCL, 4 months

#### 3.3.2. Balancing Reassurance and Uncertainty in SCID NBS Result Communication

Parents of babies later diagnosed with non-SCID TCL, described their experience being compounded by false reassurance about the likelihood of the initial NBS result being a false positive.

“…the doctor was like, ‘It’s probably nothing to worry about.’ Maybe don’t say that. Because I literally went home thinking, ‘Everything’s going to be okay.’ And it wasn’t.”

P59, Mum, Site 1, Non-SCID TCL, 8 months

Some parents reported that, although they were shocked by the initial positive NBS result, the information given during the telephone call was very helpful, reassuring and partially alleviated their fears. Other parents reported that the volume and technical nature of the information given at the time of the initial positive NBS result for SCID was overwhelming. To prevent them from accessing inaccurate information online and to support their understanding, they felt that clearer guidance and trusted resources would be helpful. Only one parent (P75 (Dad) SCID) recalled receiving a leaflet after receiving the positive SCID NBS result. Other parents felt providing leaflets and links to trusted websites would help them process and understand the information more effectively in preparation for their appointment for confirmatory testing the next day.

“…leaflets would be a massive help. Because sometimes, I can go to appointments…you’re using all these big words, it goes in one ear and out the other…leaflets or websites-, somewhere you can reread the information that you’ve just been told…it’s a lot of information to obtain.”

P73, Mum, Site 1, Non-SCID TCL, 3 months

#### 3.3.3. Awaiting Confirmatory Testing and the Search for Answers

In all cases, parents were invited to attend confirmatory testing the following day, meaning there was less than 24 h between receiving the initial positive NBS result and waiting for confirmatory testing. However, parents of babies later diagnosed with non-SCID TCL described the wait to see the immunology team as feeling much longer than it actually was.

This was made worse by being unable to sleep as parents awaited confirmatory testing, reflecting the profound anxiety and fear they experienced.

For one parent of a baby later diagnosed with SCID, the wait being so short in real terms only served to emphasize the seriousness of the potential outcome of the confirmatory testing.

“I realised that it must be something fairly serious for the doctor to ring me from the hospital to need to see him the next day.”

P45, Mum, Site 4, SCID, 28 months

All parents reported being advised not to search online before seeing the specialist immunology team for confirmatory testing. One parent felt that this heightened their anxiety, as it made them worry that something might be seriously wrong with their baby.

Parents with babies in both diagnostic groups sought information from SCID-specific sites as well search engines such as Google to ensure the information they had was reliable. For some parents, this provided useful information prior to meeting the immunology team and helped to alleviate some of their fears.

However, parents of babies later diagnosed with SCID described feeling that the information they found online only served to heighten their anxiety further and question how to care for their baby while waiting to be seen by the immunology team for confirmatory testing.

“I didn’t know what to do with [baby] because if you read it, you know, I was thinking am I allowed to touch [baby], can I hold [baby]? I just left [baby] in their cot because [baby] was asleep, and I thought…am I allowed to be near you?”

P45, Mum, Site 4, SCID, 28 months

#### 3.3.4. Reassessing Severity in the Context of a Non-SCID TCL Outcome

Parents in the present study who received a non-SCID TCL diagnosis following SCID screening assessed the severity of the outcome based on their perception that, although their baby had an immunological condition, it was not as serious as a SCID diagnosis. In this scenario, parents described a sense of relief and gratitude.

“…the overriding feeling was just pure relief that it was not SCID…. not as serious as if it was someone with SCID.”

P68, Dad, Site 6, Non-SCID TCL, 3 months

However, relief that their baby did not have SCID was quickly replaced with fear of the unknown; some parents described being in a state of limbo while waiting to find out the cause of their baby’s immunological condition and therefore future treatment plans.

Parents who received a SCID outcome described an immediate sense of shock and emotional paralysis upon receiving the news, with some recalling feeling as though their life had abruptly stopped. This initial reaction was often accompanied by deep fear and uncertainty about how to interact with their baby and fear for the future.

These parents also described immediately considering potential treatment options and associated morbidity and mortality, which led to feelings of fear and devastation.

Parents who had received either an SCID or a non-SCID TCL outcome described a cascade of thoughts as they tried to rapidly assess the potential practical and familial implications of the result. This could include catastrophising and considering how various aspects of lives could be impacted by the outcome following NBS for SCID.

“…what am I going to do about work, what’s [sibling] going to be doing, what’s, yeah, is [baby] going to be alright…basically every implication just like running through your mind, just trying to process what is going to happen next.”

P46, Dad, Site 4, SCID, 28 months

#### 3.3.5. Parenting in Protective Mode

Parents of babies diagnosed with SCID reported adopting defensive behaviours, including social withdrawal and restricting daily activities, as they sought to minimize any potential risk to their baby following the SCID NBS outcome. This had the potential to impact on several areas of their lives including work, social, and family life.

Parents of babies diagnosed with non-SCID TCL following SCID NBS described struggling to enjoy their initial experience of parenthood due to persistent uncertainty about their baby’s health. One alluded to the perceived need for a restricted daily routine and heightened vigilance to protect their baby, highlighting the emotional and social impact of caring for a baby they perceived to be at risk of infection.

“My life with [baby] is the school run, go to [supermarket], and then I walk around the park…It is horrible, really.…I hate it when my friends’ kids touch him…I wish I could just say, ‘Get your dirty little hands off him’…Because people don’t understand it… if I did go into a shopping centre, I would put his rain cover over him just so no one could actually get to him… I feel like this whole maternity being off, it’s gone that quick, and our lives were literally like hospital.”

P59, Mum, Site 1, Non-SCID TCL, 8 months

Another parent also reported the need to isolate their baby, whether that be required or self-imposed as impacting their mental well-being. It was felt that this had the potential to affect the parent who was at home caring for the affected baby more than other members of the family.

“…it’s definitely affected my mental health 100%. I would say more so me than my partner’s because…I’m here 24/7 just me and my thoughts, you know what I mean, on my own…[my] mental health I would say is poor at the minute…it does weigh you down eventually.”

P74, Mum, Site 2, Non-SCID TCL, 4 months

#### 3.3.6. Life After SCID NBS: Reassured but Still Cautious

All parents spoke very highly of their experience with the immunology teams that were involved in their baby’s initial and ongoing care. They expressed deep gratitude describing the care their baby had received as life changing.

“My experience overall was so positive. I cannot fault [hospital], and the research team. Like, I feel like I owe them something because they were just sensational.”

P73, Mum, Site 1, Non-SCID TCL, 3 months

Parents in the present study had experienced a range of treatment plans for their baby, including HSCT (SCID), thymus transplantation, and more conservative approaches such as genetic testing and regular immunology follow-ups to monitor T cells (non-SCID TCL). Parents whose baby underwent HSCT or thymus transplantation, which involved intensive treatment, potentially lengthy hospital stays, and close contact with specialist teams, reflected on the challenges of leaving the security of the clinicians they had come to trust to keep their baby safe.

“…then we got back to [home location], I found that a little bit tricky because I’d got so used to the team in [site offering transplant] being there, [baby] being protected on the ward, that then to come back and take him back to [home location hospital].”

P45, Mum, Site 4, SCID, 28 months

This was exacerbated by a perception that healthcare professionals outside the immunology team did not have the knowledge required to care for a baby with either a SCID or a non-SCID TCL outcome in the same way

“…you go to your GP, and they’ve never heard of anything to do with SCID…I had my eight-week check after I had [Baby], and the doctor had said…’We don’t know much about it at all.”

P59, Mum, Site 1, Non-SCID TCL, 8 months

Other parents were empathetic to and appreciated the non-specialist role of other healthcare professionals.

One parent of a baby diagnosed with SCID following NBS who had received HSCT described a sense of complete reassurance and how they now viewed their baby as healthy.

“…he was the first baby to have the heel prick and have the bone marrow at such a young age… and now he is a perfectly healthy, happy baby.”

P45, Mum, Site 4, SCID, 28 months

Other parents of babies with non-SCID TCL spoke of their concern decreasing over time but being ignited by regular follow-up with the immunology team.

“It’s when the appointments are approaching, that’s when my anxiety starts creeping up on me, because I’m trying not to think about it in between, and then I get an appointment and I’m, like, ‘Oh my god.’”

P74, Mum, Site 2, Non-SCID TCL, 4 months

#### 3.3.7. Impact on Families: Relationships and Resources

Parents who had received a non-SCID TCL outcome following NBS for SCID, discussed how the genetic implications of the result had impacted their relationship. One parent reported experiencing feelings of guilt and looking to apportion blame.

“…I remember feeling, like, ‘This is your fault.’ Even though I knew it wasn’t his fault, and it was something way beyond our control, I remember-, not hating [husband], but essentially hating [husband], thinking, ‘This is your fault.’…it was a lot of hatred.”

P73, Mum, Site 1, Non-SCID TCL, 3 months

Conversely, another parent of a baby with a non-SCID TCL diagnosis described how their experience of NBS for SCID had brought them closer together and highlighted how well they worked as a team.

Parents of babies in both diagnostic groups also spoke of the impact the diagnosis had or could potentially have on siblings of the affected baby. Some parents described disruptions to family systems caused by, for instance, leaving existing children with other family members while they attended appointments with the affected baby and associated concerns that their other child might feel ‘left out’.

“…leaving [sibling] was heartbreaking. There was no way [dad] wasn’t going to not be with [baby] and me…but that meant sacrificing [sibling] for we didn’t really know how long at that point.”

P45, Mum, Site 4, SCID, 28 months

Another parent described feeling conflicted about minimizing infection risk for their baby with a non-SCID TCL outcome while not restricting the older sibling’s normal activities.

“I was even thinking about taking [older sibling] out of nursery because [older sibling] is picking up all these illnesses and bringing them home and I’m, like, ‘Am I doing the best thing for her?’ But then I feel like I’m holding [older sibling) back as well if I take her out of nursery.”

P74, Mum, Site 2, Non-SCID TCL, 4 months

One parent of a baby with SCID also considered the impact of the diagnosis on their older child if they were identified as a potential donor for a HSCT for the affected baby.

“…one of the things that maybe hit me most was…it’s likely that we’d want [sibling] to be the donor for [baby], and at the time, [sibling’s] three. That was a bit upsetting really, ‘cause like, it’s just having an impact on a three-year-old as well.”

P46, Dad, Site 4, SCID, 28 months

Parents of babies who had received a non-SCID TCL outcome following NBS expressed how the result had the potential to influence parental reproductive decision-making. Parents appeared reluctant to have another baby due to the risk of having the same experience again.

Financial impacts associated with the NBS experience were also discussed by parents from both diagnostic groups. Some parents were required to travel long distances at short notice for confirmatory testing. In addition, lengthy treatment plans often necessitated extended periods away from home, during which parents had to secure alternative accommodation. Parents also expressed concern about the potential impact on their employment.

#### 3.3.8. Supporting Parents Through Their SCID NBS Journey

Parents of babies with SCID were aware that, prior to the introduction of SCID NBS, the condition was either identified (prenatally or at birth) because of a known family history or after the baby developed clinical symptoms, and that diagnosis following symptomatic onset often resulted in serious illness or even death. As a result, parents reported that much of the information available online, such as in parent forums, provided a very negative view of SCID, which did not resonate with their own experiences.

These parents were keen to see the differences between cases where SCID had been detected via family history and NBS to help them have an appreciation of what the long-term outlook might be for their baby.

“…there’s been other babies through a family history. But there wasn’t really any positive examples for me to go to and think actually [baby] might stand a good chance here, there might be complications but look, this one went to university, this one’s gone on to have a family, this baby…my thought was, will he even go to school and nursery? At this point, I’m down to will he live?”

P45, Mum, Site 4, SCID, 28 months

Parents of babies who had received a non-SCID TCL diagnosis spoke about the importance of hearing other people’s SCID NBS journeys to enable them to prepare themselves for what the future may hold.

“…just hearing what other parents had gone through when their baby had had something similar, I found quite useful because it allows you to prepare a bit.”

P68, Dad, Site 6, Non-SCID TCL, 3 months

Consequently, parents of babies with SCID and non-SCID TCL diagnoses felt it important to ensure that other parents whose babies were screened for SCID could access real-life stories that illustrated the positive impact of early detection through NBS. They emphasized the importance of appropriate resources for parents whose experiences differed from those of parents of non-screened babies with SCID. One parent of a baby with SCID also felt there needed to be an appreciation of the impact of the timing of NBS, i.e., early in the postnatal period, on parental support needs.

#### 3.3.9. Parents’ Initial Views of NBS for SCID

Even though parents of babies diagnosed with SCID had found the NBS process worrisome, they were grateful that their baby’s condition had been detected via NBS and they appreciated the importance of early detection to improve long-term outcomes for their baby.

“…relieved that it was picked up early…thankful that she was born in that two-year [SCID evaluation]…I would much rather them put me and [mum] through 24 h of crap to then be told everything’s fine than the chance that we don’t find out and then we lose our baby in two years because that wasn’t an option to be tested for.”

P68, Dad, Site 6, Non-SCID TCL, 3 months

One parent of a baby with SCID described experiencing feelings of ‘*survivor’s guilt*’ and how this manifested when speaking with other parents whose baby had been diagnosed clinically.

“I connect with these families, and I see the suffering that they have and I feel guilt. I have a sense of, like, survivor’s guilt…why did [baby] get the heel prick test, why is he allowed to have the heel prick test and these other babies weren’t?”

P45, Mum, Site 4, SCID, 28 months

For parents in both diagnostic outcome groups, the NBS experience was associated with a shift in perspective as they reported a greater appreciation for what they viewed as truly important, a reduced focus on everyday worries and appreciation of the opportunity for their child to be screened for SCID.

“I got given a golden ticket when it came…[Baby] got given a golden ticket when it came to the NHS because there wasn’t a single day where [Baby] was not put at the centre…”

P46, Dad, Site 4, SCID, 28 months

### 3.4. Themes: Follow up Interviews with Parents Following Their SCID NBS Journey

Six follow-up interviews were undertaken. Five were single interviews with two mothers (P64 and P74) and one father (P68) of babies with a non-SCID TCL outcome, and one mother (P45) and one father (P46) of a baby with a SCID outcome. The sixth was a joint interview with the mother (P18) and father (P19) of a baby with a SCID outcome. Ages of babies at the time of the follow-up interviews ranged from 12 to 41 months (a median 13 months).

#### 3.4.1. The Usual Case of SCID via NBS

One parent of a baby diagnosed with SCID following NBS described the difficulty of reconciling their experiences over time, initially viewing their baby as healthy, then learning through NBS that their baby had a serious medical condition and finally being told post-treatment that their baby was healthy again. Despite this, they expressed that they could not forget the extent of the treatment their baby had undergone and held an underlying concern about the potential long-term impact.

The father of this baby also described the experience of attending a multidisciplinary meeting where numerous clinicians from different hospitals gathered to discuss their baby’s case. They reflected on the overwhelming nature of being surrounded by so many professionals who had taken a deep interest in their baby’s care, which was a stark contrast to their otherwise ordinary day-to-day life. This encounter highlighted the additional attention their baby was receiving and served as a poignant reminder of the significance of his medical journey.

“…you go into this room and it’s like people from [Hospital A] and the [Hospital B] team, we had so many people, I couldn’t take it in. All the people from [Hospital C] who wanted to come and see him and be part of the conversation and we sat in the middle and we’re surrounded like surrounded by 7–8 people all just wanting to listen in about him…We just live a normal life and I’m more of a like, forget that, that kind of like, you know, part that we’ve moved on. But it kind of shocks you because you’re like wow, [son]’s drawing in this this kind of crowd from across the country…it kind of brings it all back, the sort of significance of it all, I suppose…It was like a bit overwhelming, all these people are just here to check in on [son] and he’s just a normal boy in my eyes I know…there’s just a big contrast to like our life for the other 364 days of the year.”

P46, Dad, Site 4, SCID, 41 months

Another parent, whose baby had been diagnosed with a non-SCID TCL following newborn screening for SCID, described trying not to be overly cautious about exposure to potential infections. Over time, their fears had been somewhat allayed as their baby recovered from minor infections they had been exposed to.

“…now, where we’ve seen her get sick, we’ve seen her recover really well. We’ve seen her just get on with things…ultimately, I’m not as nervous or cautious with her anymore.”

P68, Dad, Site 6, Non-SCID TCL, 12 months

A parent whose baby had received a SCID diagnosis following a positive NBS result, reflected on the emotional toll the experience had taken on their mental health. This parent described the emotional extremes of being told, within a short space of time, that their baby had a life-threatening condition, followed by the message that their baby was “healthy” following treatment, a shift they found particularly difficult to process and reconcile.

“You kind of wish that that would be recognized about what you’ve been through and not just be classified as normal. Some doctors would walk in the room and just be like it’s normal now. What are you doing here? Is he sick? He’ll get sick and he’ll get better…everyone’s extremely positive about him, but there is always a clause of, but you know he has got SCID. We’ve not changed, [son], we’ve given him a transplant and that transplant hopefully will live with [son] for a very long time. But my understanding is we’re not cured.”

P45, Mum, Site 4, SCID, 41 months

This baby’s father reflected on the transition from hospital to home following their baby’s transplant, highlighting the challenges of adapting to significantly reduced support compared to the structured and readily available care provided within the hospital setting.

The mother of this baby also spoke about these feelings subsiding over time and, therefore, their need for additional support also reducing.

For one parent of a baby diagnosed with non-SCID TCL, the feelings of guilt were associated with conditions identified via SCID screening being genetic and coming to terms with the fact that if their baby were found to have the condition, it would have been inherited from them.

“It was just the torture of you putting yourself through it by trying to know everything and because of the nature…the genetic condition, the tests were also on myself and my partner to see if it’s something that she inherited from us.”

P68, Dad, Site 6, Non-SCID TCL, 12 months

#### 3.4.2. Communication and Information Provision About NBS for SCID

Parents who had been through the journey of receiving a positive NBS result and whose baby had been identified with a non-SCID TCL appreciated the complexity of the different outcomes and therefore the difficulties of explaining this at the point of obtaining the NBS sample.

Making parents aware of the possible outcomes of NBS, either antenatally or at the time of NBS, was therefore considered important. This was felt to be particularly relevant for SCID NBS, where the false positive rate may be higher than some of the other conditions included in the NBS Programme.

“I do think it would be good for parents to know that again as well, that there is a high chance of a false positive as well.”

P74, Mum, Site 2, Non-SCID TCL, 13 months

It was also felt important to highlight that NBS for SCID could also detect other immunological conditions that may also require monitoring and/or treatment.

“I don’t think it was truly ever outlined to me that if it’s not, if this test comes back positive, it could also mean 50 other things.”

P68, Dad, Site 6, Non-SCID TCL, 12 months

Parents reiterated again during the follow up interviews that if insufficient information is provided about the NBS process, parents will use online search engines to fill in the gaps. This poses risks in terms of them accessing information that is not relevant but may heighten their concern.

Although communication of the positive NBS result was distressing for parents, in hindsight, one parent of a baby with SCID recognized that it may not be possible to deliver the result in a way that is not going to be worrisome for parents.

During the follow-up interviews, parents of babies diagnosed with both SCID and non-SCID TCL, continued to speak very positively about their interactions with the immunology teams, particularly in relation to the information provided and the reassuring qualities of team members, which helped alleviate their concerns.

“The support that we got from [hospital], I couldn’t thank them higher [sic]. They were very clear on the timelines. They were very clear on what each step’s going to be.”

P68, Dad, Site 6, Non-SCID TCL, 12 months

Similar to the initial interviews, parents felt the timeliness of confirmatory testing and the speed at which the diagnostic outcome became available were appropriate and had helped them through their SCID NBS journey.

#### 3.4.3. Parents Long-Term Views of NBS for SCID

During the follow-up interviews, parents of babies with both diagnostic outcomes reflected that, despite the emotional and practical challenges, their NBS and treatment journeys were ultimately worthwhile, given their baby’s positive outcome.

Parent felt that NBS for SCID provided critical early identification of their baby’s condition, enabling proactive care and reducing the risk of potential complications in the future, which they felt would not have been possible otherwise.

These feelings were intensified by an awareness of other families whose baby, in the absence of early screening and intervention, experienced more serious illness and endured more challenging diagnostic and treatment pathways.

“When I speak to sort of the other SCID parents, they’re quite angry that they didn’t have a screening test. It feels a bit, a bit like guilty. Sometimes, like my baby’s really well and yours isn’t. And it’s a different conversation. We’ve both been through transplant, but in very different ways.”

P45, Mum, Site 4, SCID, 41 months

Overall, parents whose babies had been diagnosed with a condition via NBS were extremely grateful that they had been part of the SCID evaluation and the perceived positive impact it had had on their baby but also their families’ health and well-being.

“I always say that I could give me £1M or screening and I could always take the screening, like always, because [son] would not be the way he is today… I live with the positive impact of the screening.”

P46, Dad, Site 4, SCID, 41 months

## 4. Discussion

Comparing experiences of parents whose baby had received a SCID or non-SCID TCL diagnosis as part of the SCID NBS evaluation in England allowed for a nuanced understanding of how perceived diagnosis severity, uncertainty, and treatment urgency shaped parental experiences, coping strategies, and support needs.

Parents of babies in both diagnostic groups recalled a range of negative emotions associated with receiving the initial positive NBS result for SCID during the interviews. This included devastation, shock, grief, fear, and a feeling that their world had fallen apart. This reflects the findings of previous studies that have explored parental reactions to the positive NBS result for a variety of conditions including cystic fibrosis and a range of metabolic conditions [17,18,21,22,23,24]. Similar to previous studies [17,18,21,22,23,24], parents continued to have vivid recollections of being informed of their baby’s positive NBS result during the follow-up interviews. However, with the benefit of hindsight, parents whose babies had received a SCID diagnosis appreciated that it was probably not possible to deliver this information in a manner that did not cause some level of anxiety for parents.

Before receiving the positive NBS result for SCID, parents often had no inclination that their baby could have an underlying health condition; parents had to reconcile the positive NBS result with their seemingly ‘healthy’ baby. Indeed, the purpose of NBS is to identify rare but serious medical conditions pre-symptomatically and refer them for urgent confirmatory testing and, if necessary, treatment [25]. This could make parents question their parenting abilities due to them not having recognized that there could be something wrong with their baby.

The emotional toll of receiving a positive SCID NBS result was evident. During the evaluation, two-thirds of babies born in England were offered screening, while the remainder comprised a comparator group. Due to this, one parent whose baby had been diagnosed with SCID via NBS spoke of ‘survivor’s guilt’ when they compared themselves to families for whom screening was not available (either because they were born before the SCID NBS evaluation or were part of the comparator group) and whose baby had been diagnosed with SCID due to being symptomatic or having a family history. Feelings of guilt associated with a positive NBS result [17,26] and passing on faulty genes to offspring [27] have been reported previously in the literature. However, parental guilt associated with the opportunity to have their baby screened has not been reported elsewhere. In the present study, parents perceived families for whom screening was not available, as experiencing protracted diagnostic journeys and poorer outcomes. This has also been reflected in the literature in relation to inborn errors of immunity in their entirety rather than SCID specifically [28].

Parents of babies diagnosed with non-SCID TCL viewed their baby’s result in terms of relative risk compared with a SCID diagnosis. While their baby might still have a serious immunological condition, parents did not consider this to be as serious as SCID. This may have reflected the different treatment pathways following a SCID or non-SCID TCL diagnosis and the projected outcomes. While treatment for babies with SCID would necessitate strict isolation, infection control precautions for babies with a non-SCID TCL diagnosis depends on the severity of their immunodeficiency. Despite this, these parents spoke about taking extra precautions to prevent their baby being exposed to what they considered unnecessary risks, which commonly resulted in reduced socialization that included delaying sending their baby to nursery, reducing trips outside the home and reducing contact with friends and family.

Some parents were concerned with how the SCID or non-SCID TCL result might affect their relationship with their unaffected children. It is known that siblings of children with a chronic illness can experience depression, anxiety and negative self-concept [29,30]. Therefore, careful consideration should be given to how to support the wider family during the first few years following the baby’s diagnosis.

Parents had mixed feelings about how the NBS result for SCID had impacted their relationship. One parent whose baby had received a non-SCID TCL diagnosis spoke about the experience making their relationship stronger and bringing them closer together. Another parent of a baby with the same outcome spoke of the negative impacts associated with the genetic component of the diagnosis and attribution of blame. While it was not clear from the interviews why this difference existed, this may have been attributable to parents reflecting on different aspects of their SCID NBS journey. Following the initial positive SCID NBS result, parents may have reflected on the potentially life-threatening outcome if their baby had been diagnosed with SCID which could have united parents and strengthened their relationship, whereas once the non-SCID TCL outcome was known, genetic uncertainty may have contributed to stress and negative impacts on parental relationships. This reflects the previous literature that has explored the impact of uncertainty following NBS for cystic fibrosis [24,31].

Parents whose babies had a non-SCID TCL result discussed the implications of their baby’s result on their future pregnancies. This reflects previous research with parents following a positive NBS result for various conditions included in NBS in terms of parents being more cautious about having future babies due to guilt associated with passing on a faulty gene but also the implications for the baby’s future [32,33].

There were limited support communities for parents who had been through SCID NBS, particularly for those whose baby received a SCID diagnosis. Existing stories and support groups were mainly focused on diagnosis following clinical presentation or family history. This had the potential to leave parents who had been through SCID NBS feeling isolated and with few opportunities to share experiences or seek guidance from others in similar situations.

All parents consistently expressed positive views of the clinical teams involved in diagnosis and management (during both the initial and follow-up interviews). Following intensive treatment regimes, parents of babies with SCID described challenges in adjusting to reduced contact with the clinical team after their baby’s treatment.

Parents of babies diagnosed with SCID and non-SCID TCL expressed concerns about support from the wider healthcare team mainly due to their perceived lack of knowledge of the condition. This might become even more important if the SCID NBS programme is rolled out across the UK which may require the input of various non-specialists (e.g., General Pediatricians) to help deliver the programme. Participants valued timely appointments, clear explanations, and the empathy shown by the immunology teams. In particular, the responsiveness of immunology consultants and the efficiency of confirmatory testing were repeatedly highlighted as providing reassurance during what was perceived as an extremely challenging time. As SCID is considered a rare condition, familiarity with its management may be limited not only among non-specialists but also among immunologists outside specialist centres. Healthcare professionals outside these specialist centres (including immunologists) may be more focused on infectious diseases rather than immunology; while they possess the necessary medical knowledge, direct clinical experience with SCID cases may be limited. The importance of knowledgeable health professionals who are able to answer parental questions and provide reassurance during the NBS process has been highlighted previously [18].

Regardless of the NBS confirmatory test outcomes, all parents considered NBS to be important and felt grateful for the opportunity to have their baby screened for SCID.

Strengths and limitations: Recruiting parents with a positive NBS result that resulted in a diagnosis of SCID or non-SCID TCL was challenging, given that the first year following their baby’s confirmatory testing result could consist of complex treatment regimens and/or numerous hospital appointments, which meant that they were reluctant to be interviewed until their baby was slightly older. Consequently, recruitment was low—25% of babies detected with SCID and 11% of babies who received a result indicating their baby had a non-SCID TCL—which resulted in a small overall sample size and, in particular, parents of only three babies diagnosed with SCID. The timing of interviews also differed between the SCID and non-SCID TCL groups, which may have influenced parental recall and therefore the interpretation of their experiences. In addition, the interview sample was largely composed of parents identifying as English/Welsh/Scottish/Northern Irish/British and English-speaking. However, the SCID population includes a high proportion of families from minoritised ethic groups and/or where English is not their first language. Given that the parents in the current study reported challenges navigating the SCID NBS journey, parents from underrepresented ethnic groups or those with additional linguistic or cultural barriers may experience even greater challenges in accessing information and understanding NBS results. The interviews were in-depth, and follow-up interviews allowed for the extensive exploration of issues discussed.

## 5. Conclusions

There were many similarities and some nuanced differences in parents’ experiences following SCID NBS for babies later diagnosed with SCID and non-SCID TCL. All parents described shock at receiving an initial positive NBS result. For parents of babies with SCID, this shock was linked to previously unknown genetic risk, whereas for those with a baby with non-SCID TCL, it reflected the absence of symptoms in their infant. Parents of babies with non-SCID TCL generally perceived the outcome as less severe than SCID but continued to experience anxiety related to diagnostic uncertainty.

Although parents found the information provided by immunology teams helpful, they all sought additional information online, which increased anxiety, particularly among parents of babies with SCID. Across both groups, parents perceived limited knowledge of SCID and non-SCID TCL among healthcare professionals outside specialist immunology services.

Parents of babies with SCID described the required period of isolation prior to treatment as particularly challenging, while those with a baby with non-SCID TCL reported self-imposed isolation driven by fear. Parents in both groups also described the impact of the diagnosis on siblings, including separation during treatment, and concerns about potential HSCT donation in SCID. The impact on reproductive decision-making for parents of babies with non-SCID TCL was also discussed.

Parents in both diagnostic groups emphasized the value of peer support from families with similar NBS experiences, with support needs reflecting the differing clinical and emotional trajectories of SCID and non-SCID outcomes. Consequently, support for parents following a diagnosis of SCID via NBS requires careful consideration, as their experiences may not compare with those of parents whose baby has been diagnosed due to family history or clinical symptoms.

Based on their experiences, all parents would recommend that NBS for SCID be included in the national NBS Programme in England. However, the evaluation in England found that SCID NBS was not cost-effective and will therefore continue as an in-service evaluation at present [34].

## Figures and Tables

**Table 1:** Ages of babies at first and follow-up interviews and duration by NBS outcome.

Category ^1^	Subtype	Site	Identifier	Age of Baby Initial Interview (Months)	Duration of Initial Interview (Minutes)	Age of Baby Follow-Up Interview (Months)	Duration of Follow-Up Interview (Minutes)
SCID	-	5	P18 (mother)	13	106 (joint P19)	25	57 (joint P19)
SCID	-		P19 (father)	13	106 (joint P18)	25	57 (joint P18)
SCID	-	4	P45 (mother) &	28	92	41	38
SCID	-		P46 (father)	28	74	41	37
SCID	-	2	P75 (father)	20	40	-	-
Non-SCID TCL	2p11.2 deletion	1	P59 (mother)	8	45	-	-
Non-SCID TCL	unspecified	3	P64 (mother)	4	57	13	25
Non-SCID TCL	DiGeorge Syndrome	6	P68 (father)	3	55	12	30
Non-SCID TCL	unspecified	2	P71 (mother)	20	51 (joint P72)	-	-
Non-SCID TCL	unspecified		P72 (father)	20	51 (joint P71)	-	-
Non-SCID TCL	unspecified deletion	1	P73 (mother)	3	54	-	-
Non-SCID TCL	unspecified deletion	2	P74 (mother)	4	45	13	21

^1^ Non-SCID TCL = non-SCID T-cell lymphopenia.

## Data Availability

The data are not publicly available due to ethical constraints.

## Abbreviations

The following abbreviations are used in this manuscript:

CF	Cystic Fibrosis
HSCT	Hematopoietic Stem Cell Transplantation
NBS	Newborn Screening
NHS	National Health Service
PIS	Participant Information Sheet
SCID	Severe Combined Immunodeficiency
TRECs	T-lymphocyte Receptor Excision Circles
UKNSC	UK National Screening Committee
